# The Clinical Researcher Journey in the Artificial Intelligence Era: The PAC-MAN’s Challenge

**DOI:** 10.3390/healthcare11070975

**Published:** 2023-03-29

**Authors:** Elena Giovanna Bignami, Alessandro Vittori, Roberto Lanza, Christian Compagnone, Marco Cascella, Valentina Bellini

**Affiliations:** 1 Anesthesiology, Critical Care and Pain Medicine Division, Department of Medicine and Surgery, University of Parma, Viale Gramsci 14, 43126 Parma, Italy; 2 Department of Anesthesia and Critical Care, ARCO ROMA, Ospedale Pediatrico Bambino Gesù IRCCS, Piazza S. Onofrio 4, 00165 Rome, Italy; 3 Department of Anesthesia and Critical Care, Istituto Nazionale Tumori—IRCCS, Fondazione Pascale, 80131 Naples, Italy

**Keywords:** artificial intelligence, research, anesthesia, intensive care, pain, hospital, big data, legal medicine, data analysis, education

## Abstract

Artificial intelligence (AI) is a powerful tool that can assist researchers and clinicians in various settings. However, like any technology, it must be used with caution and awareness as there are numerous potential pitfalls. To provide a creative analogy, we have likened research to the PAC-MAN classic arcade video game. Just as the protagonist of the game is constantly seeking data, researchers are constantly seeking information that must be acquired and managed within the constraints of the research rules. In our analogy, the obstacles that researchers face are represented by “ghosts”, which symbolize major ethical concerns, low-quality data, legal issues, and educational challenges. In short, clinical researchers need to meticulously collect and analyze data from various sources, often navigating through intricate and nuanced challenges to ensure that the data they obtain are both precise and pertinent to their research inquiry. Reflecting on this analogy can foster a deeper comprehension of the significance of employing AI and other powerful technologies with heightened awareness and attentiveness.

## 1. Introduction

Clinical researchers are professionals who plan, design, and conduct clinical studies to advance scientific discoveries while ensuring that ethical principles and standards are upheld [1]. In recent years, the increasing use of new technologies, particularly artificial intelligence (AI) [2], has greatly enhanced the potential of these tools for clinical research. AI has demonstrated impressive results in every phase and type of study, including drug discovery, protocol optimization, clinical trials, and data management [3,4].

In particular, the capability to handle extensive volumes of data provides researchers rapidly and precisely with unprecedented prospects to achieve substantial breakthroughs that were previously deemed unattainable [5]. Consequently, the rapid evolution of new technologies has revolutionized the field of clinical research, offering exciting opportunities for researchers to improve the accuracy and efficiency of their work. However, these advancements also come with significant challenges that must be necessarily addressed to ensure their successful implementation [6]

One of the most critical challenges facing clinical researchers is the need for a high level of awareness and understanding of these new technologies. While they offer a wide range of benefits, they can also lead to potentially serious errors if not used correctly. This requires researchers to be well-versed in all aspects of the technology, including its strengths, weaknesses, and limitations.

To fully capitalize on these tools, clinical researchers must be motivated to continuously acquire knowledge and develop practical solutions that can help them address complex research questions. This means staying up to date on the latest advancements, collaborating with experts in other fields, and constantly seeking new approaches to improve their work [7].

Despite the challenges, the benefits of using new technologies in clinical research are undeniable. For instance, through their capability to process large amounts of data quickly and accurately, they can offer researchers the opportunity to make significant breakthroughs that were once thought impossible. By utilizing these advancements efficiently, clinical researchers can expand the frontiers of what can be achieved and unveil novel insights that have the potential to revolutionize healthcare practices [8].

Drawing an imaginative analogy, we compared the role of a clinical researcher to that of the PAC-MAN video game protagonist, whose insatiable appetite for dots and energy pills is akin to the clinical researcher’s unrelenting pursuit of knowledge and considerable scientific objectives. Following this parallel, we have developed a simple guide for helping those working with AI technologies in the anesthesiology field. It should be useful to optimize the creation, development, and evaluation of research projects.

## 2. The PAC-MAN Metaphor

PAC-MAN is a classic arcade video game that gained immense popularity in the 1980s. Initially, “Puck Man” was created by the Japanese video game developer and publisher Namco. Midway Games later obtained the license to distribute the game in the United States and changed its name to “PAC-MAN”.

The game features a comical round yellow character who must collect as many yellow balls or pellets as possible while navigating through a maze [9]. Throughout the game, various elements (fruits) appear at random intervals, providing PAC-MAN with extra bonuses that help him achieve his objective more quickly [10].

**Figure 1:**
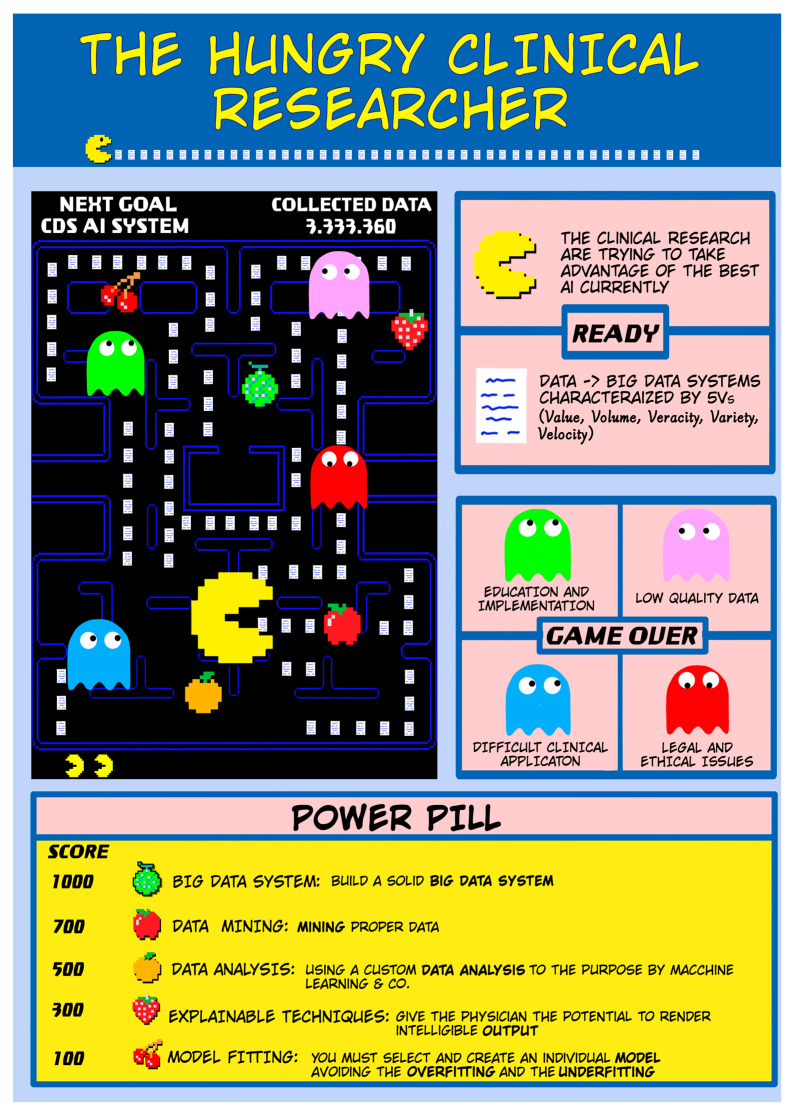
The Hungry Clinical Researcher. It is a metaphor for a clinical researcher’s job and shows the tools, objectives, and open issues of clinical research in AI.

The metaphor of the hungry clinical researcher reflects the complexity and challenges of conducting research in AI. It highlights the need for multiple tools and resources, the importance of collaboration across disciplines, and the ongoing efforts to address open issues and maximize the benefits of AI in healthcare (Figure 1).

In the context of clinical research, the “yellow balls” can be likened to data, which are the essential building blocks for creating an effective and robust study.

The “fruits” are intermediate steps to accomplish the objective; once present, they can give stability to the methodology and thus allow for reaching the goal. These are represented by: 

*Building a big data system*. A big data system pertains to a technological framework engineered to store, process, and analyze vast amounts of data that may be unstructured, heterogeneous, and produced at great speed. Building a big data system entails a sequence of phases that mandate careful planning, proficiency in data science technologies, and a profound comprehension of the objectives and data prerequisites [1].

*Mining proper data, extracting, and recognizing useful information*. An essential task in data analysis is to identify pertinent data from a vast collection of available data, and apply diverse tools and techniques to extract valuable insights and knowledge.

Using a custom data analysis to achieve the purpose. This entails tailoring data analysis techniques and tools to the specific needs and objectives of a research project. This process enables researchers and analysts to extract more relevant and valuable insights [11].

*Rendering intelligible output*. This is the process of transforming complex or abstract data into a form that is easily understood and interpreted by other researchers [2].

*Selecting and creating an optimized model*. This refers to the process of choosing the best algorithm or technique for a specific data analysis task and then fine-tuning its parameters to achieve the best possible performance [12].

On the other hand, enemies are represented by all the steps that must be faced. The “ghosts” are [3]:

*Major ethical and legal issues*. While ethical and legal considerations are always present in clinical research, the use of AI raises specific issues that must be addressed. The ethical implications are so significant that, in 2018, the theologian Paolo Benanti introduced the concept of algorethics. This is defined as the study of ethics applied to technology [13,14]. Since then, many important institutions have drafted recommendations for ethical and conscious AI use, such as the United Nations Educational, Scientific and Cultural Organization (UNESCO) adopting the first global agreement on AI ethics in 2021 [15]. The concrete risk, as depicted in dystopian films and books, is that AI may replace human beings rather than serve as a valuable tool. This would have immediate implications on the labor market, as seen during the industrial revolution, and more long-term implications in terms of security. However, legal regulation on the use of AI in medicine and clinical research is still in its infancy. One of the most discussed issues is the responsibility associated with several approaches described in the literature, including the formal, technological, and compromise approaches [16]. The formal and technological approaches differ in terms of fault attribution, with responsibility falling, respectively, on the developer or the insurance company. In the case of the compromise approach, the regulation focuses solely on the ethical question. The best approach is not yet known, and likely no single answer exists. European law has introduced the rule that the “developer’s responsibility is directly proportional to the autonomy of the AI robot.” However, most authors agree that AI is not yet mature enough to have legal responsibilities, and, therefore, AI should be seen as a clinical tool with humans bearing full responsibility [17]. In summary, the main ethical and legal issues related to AI can be categorized as algorithmic transparency and accountability; data bias, fairness, and equity; data privacy and security; malicious use of AI; and informed consent to use data [15,18]. Liability for harm and cybersecurity are also important considerations.

*Low-quality data*. Large datasets are crucial for Machine Learning and Deep Learning, but the use of low-quality or irrelevant data can seriously compromise the efficiency and usability of these systems. This is commonly known as the GIGO rule in computer science, which emphasizes that “garbage in, garbage out”. This means that the quality of the output is only as good as that of the input. In healthcare, where the output can directly affect crucial clinical decisions, this rule takes on an even greater significance.

*Difficult clinical application*. The main limitations in daily practice include performance drift, lack of external validation, lack of uncertainty quantification, and lack of proven clinical value. These limitations prevent the achievement of the fifth V of big data systems, which is the clinical value derived from the application of AI in clinical research and practice. Without overcoming these limitations, AI models remain only theoretical tools that are not applicable in the real world [10]. 

*Education and implementation*. Training physicians to understand the potentials and limits of AI clinical use [19,20] and studying strategies for their implementation in daily clinical practice are essential steps to extrapolate the full potential of these tools [13,21]. The risk is either not to use them or to use them incorrectly, implying, respectively, an underuse or an error.

Notably, clinical researchers and healthcare professionals employing AI techniques need to know these issues and try to address them (Table 1). However, not all roadblocks currently have a clear and practical solution, and several studies are underway to understand the best ways to overcome current limitations.

Obviously, the PAC-MAN metaphor should be interpreted as a simplistic reading of a complex and multifaceted problem. In fact, it is not possible to approach the theme of AI like a video game. However, the PAC-MAN metaphor, like all metaphors, can also be useful in arousing interest in a new field of clinical and research application.

## 3. Game Rules for a Clinical Researcher

Like in any game, a player (i.e., a clinical researcher) must follow rules to reach the targets. One of the main rules for our protagonist, PAC-MAN, is to eat all the Pac-dots in the labyrinth while avoiding being touched by ghosts. Failure to do so results in the loss of one of the available lives. Similarly, to bring a study from theory into practice, a researcher must follow guidelines and parameters to minimize bias, ensure replicability, and enhance reliability. 

However, as AI in anesthesia is a relatively new field, there were initially no specific guidelines to follow. Existing guidelines did not fully adapt to the methodology and presentation of results generated using AI techniques, resulting in inadequate reporting and insufficiently robust studies. This could lead reviewers to draw incorrect conclusions [22,23]. Nowadays, there are various types of guidelines available, some of which are an extension of existing ones, to aid researchers in using AI in healthcare (Table 2) [23,24,25,26,27,28,29,30]. Adhering to these guidelines is critical for researchers, much like PAC-MAN following the game rules. Failure to do so could result in a progressive loss of the study’s robustness until it becomes clinically irrelevant. Following guidelines is a secure pathway to deliver high-quality output since they address specific AI issues and were developed to guide clinical research in this context [31]. Transparency in research is a novel feature that must be present and is one of the main aspects addressed by the guidelines. The clinical implications of using AI tools can be significant, and, therefore, applying the correct methodology is imperative [32].

The introduction of AI and new technologies is changing the traditional rules of the game in medicine. In recent decades, clinical practice has been founded on evidence-based medicine (EBM), which relies on the current scientific literature, individual practice, and the specific characteristics of each clinical case [33]. Although EBM is not perfect, it has become an essential part of modern medicine [33]. The disruptive force of new technologies is gradually changing this paradigm, as scientific evidence of the potential of these technologies in medicine continues to grow. These intelligent tools are increasingly entering our clinical practice, as researchers and physicians. However, their different methodology challenges the traditional pyramid of evidence, which is a cornerstone of EBM. To address this, some authors have proposed modifying the pyramid to include evidence from AI in clinical practice and medical research. This proposal would guide the evaluation of AI technologies in everyday life and help clinicians to navigate this new area of medicine. However, there is no unique game strategy for clinical researchers to follow. Just as PAC-MAN can choose different paths to tackle the maze, clinicians can reach their goals through different approaches. For example, in recent years, many predictive models have emerged that were validated internally but lacked external validation. Clinicians must consider whether it is appropriate to externally validate each proposed model before moving on to the next one, or whether there is a better approach. Ultimately, the goal should be to make the most of these technologies for our patients, and clinicians must continue to evaluate and improve their use of these tools to achieve this goal.

## 4. AI in Anesthesia

Although comparing a researcher to playing a video game may be a simplistic analogy, AI applied to clinical anesthesia requires addressing several scenarios to have a consolidated experience. Despite the term being coined in 1956 by American mathematician John McCarthy, there have been many unsuccessful attempts to carry out certain tasks in the field of anesthesia. However, recent advancements have led to the slow closing of the deep chasm between AI-based research articles and their application to clinical anesthesia.

For instance, the implementation of closed-loop systems capable of accurate risk assessment, intraoperative management, automated drug delivery, and predicting perioperative outcomes could reduce the intensity of physician work, improve efficiency, lower the rate of misdiagnosis, and lead to a fully automated anesthesia maintenance system. However, AI has its limitations and cannot replace human skills, communication, and empathy of hospital staff. Additionally, AI systems are at a preliminary and immature stage, and their auto-updating still requires human interface [34].

Clinicians must serve as administrators in governing the use of clinical AI. One example is “dataset shift”, a malfunction that occurs when an ML system underperforms due to a mismatch between the data set with which it was developed and data for which it is deployed. When using an AI system, clinicians should note any misalignment between the model’s predictions and their clinical judgment, and act accordingly [35]. However, the use of AI in healthcare may lead to new types of errors, which will require targeted strategies to study and adapt clinical practice.

The manufacturers of AI tools must declare their safety margin, and users must be aware of it. For example, Esteva et al. [36] showed that in the discrimination of benign and malignant melanocytic lesions, while human doctors overdiagnosed, the model underestimated, leading to potentially unfavorable outcomes. AI models do not consider their real implications, and users must be aware of these behaviors to limit the misuse of these tools. It is necessary to train for this new hybrid work team, transforming the use of AI tools into real collaboration, with the optimization of patient outcomes as a common goal rather than only the best predictive performance.

## 5. Clinical Practice and Research Perspectives

The ultimate goal of AI is to develop intelligent tools that can enhance our clinical practice, increase patient safety, and improve diagnostic and treatment accuracy. However, AI is currently unable to provide the same level of results in all areas of medicine due to varying interests and specializations. 

Radiology is an excellent example of how AI can improve clinical practice through the creation of intelligent tools. Conversely, other areas, such as predicting postoperative complications in perioperative medicine, have not yet produced the desired results. Although many predictive models have been published, most are still in the research stage, and a valid and universally applicable intelligent tool for clinical practice has yet to be developed [37,38,39,40,41,42,43,44,45]. 

**Figure 2:**
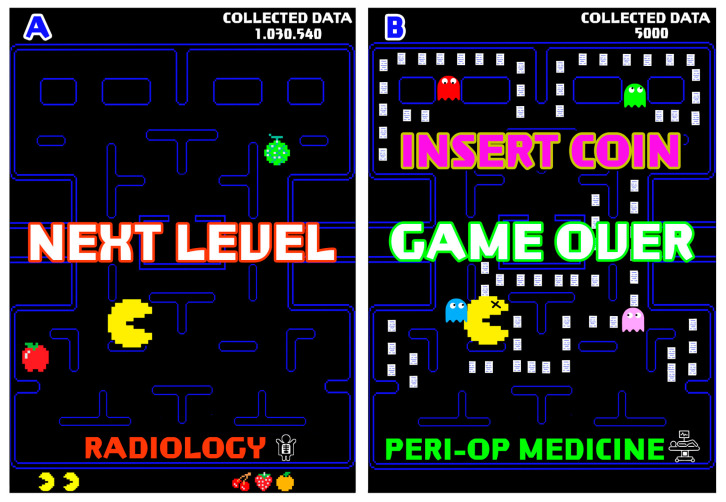
An example of AI in clinical practice. On the left side (**A**), the researcher manages to win the challenge about AI in radiology scenario (“NEXT LEVEL”). On the right side (**B**), the researcher fails to achieve the goal in perioperative medicine (“GAME OVER”), as many issues about AI are still to be solved in this context, represented by ghosts. “INSERT COIN” identifies the many challenges that AI faces in clinical practice before its widespread use.

Nevertheless, even if a model with strong performance is developed, it is not a guarantee of success. According to the game metaphor, “true victory” is only achieved if the model created is valid and applicable to daily clinical practice (Figure 2).

By following this approach, it is possible to enhance the applications of AI and explore new avenues for future research. For example, Explainable AI is an emerging research field that aims to create AI systems capable of justifying their decisions and offering insight into the reasoning behind their conclusions. This could be especially valuable in medical contexts, where comprehending the rationale behind a decision is critical [46]. Additionally, predictive analytics is an AI domain that can leverage past data to make predictions about future events. In the medical domain, predictive analytics has the potential to anticipate patient outcomes and aid healthcare practitioners in their treatment decision-making [47]. Furthermore, Natural Language Processing (NLP) is an AI field focused on enabling interaction between humans and computers using natural language. Remarkably, there is a great potential for future research to investigate how NLP can be utilized in healthcare settings to enhance communication between healthcare professionals and patients [48]. Finally, multi-modal data analysis involves combining data from different sources, such as medical images, clinical notes, and genetic data, to provide a more comprehensive picture of a patient’s health [49].

## 6. Conclusions

The PAC-MAN analogy can serve as a useful reminder of the essential role that data play in clinical research, and highlights the importance of gathering, analyzing, and utilizing data in a thoughtful and strategic manner. Just as PAC-MAN must navigate through a complex maze to collect all the dots, clinical researchers must carefully gather and analyze data from a variety of sources, often working through complex and nuanced issues to ensure that the data they collect are accurate and relevant to their research question.

In the future of healthcare, and particularly in fields such as anesthesia and critical care medicine, AI is set to continue playing a significant role, and it is crucial to adopt suitable technologies that uphold medical ethics principles. Nevertheless, while AI presents numerous benefits, it is a complex tool that requires strict adherence to precautions to prevent any potential negative outcomes. 

AI, being a completely new field of clinical and research application with possible enormous developments in the future, is open to speculation. Such speculations are dangerous, not only for those who want to automatically replace human intelligence with artificial intelligence, but, if possible, they are even more dangerous in the field of research. The human factor is fundamental in a field such as Anesthesia and Critical Care, where not everything can be reduced to science. If the human factor is eliminated from research, this will also inevitably produce disasters in the clinical field. Like any change, the advent of AI needs to be governed and addressed.

## Figures and Tables

**Table 1:** Relevant issues and main specific items.

RELEVANT ISSUES	MAIN SPECIFIC ITEMS
ETHICAL ISSUES	Could the algorithm be used maliciously? What are the possible biases in the developed system? Have techniques been used to optimize the fairness of the system?
LEGAL ISSUES	Has the issue of patient privacy been addressed? How is cybersecurity managed? Is the algorithm open or protected?
EDUCATION AND IMPLEMENTATION	Have implementation strategies in clinical practice been studied? What are the main roadblocks of the system in its possible implementation in clinical practice? How should healthcare professionals be trained to use the new system?
QUALITY OF DATA	What is the quality of exploited data? Is the quality of data collected and analyzed demonstrable? Have transparent data transformation techniques been used?
CLINICAL APPLICATION	Is the algorithm applicable in real clinical practice? Does the algorithm require particular instruments/software/digitization processes to be applied? Has the proposed algorithm been externally validated? Does the model employ interpretable AI techniques?

Legend. Relevant challenges to the use of AI techniques in medicine are reported. Next to them, some of the main specific items that are essential to developing tests or using an AI-based clinical decision support system are listed. These issues should be addressed or researched whenever an AI tool for healthcare is created or implemented in clinical practice, respectively (AI = Artificial Intelligence).

**Table 2:** The main guidelines regarding the use of AI in clinical research.

Guideline	Description	Setting
SPIRIT-AI [23]	Standard Protocol Items: Recommendations for Interventional Trials—Artificial Intelligence	This promotes transparency and completeness for clinical trial protocols for AI interventions.
CONSORT-AI [25]	Consolidated Standards of Reporting Trials—Artificial Intelligence	This was developed to supplement SPIRIT-AI in order to improve the quality of trials for AI interventions.
STARD-AI [26]	Standards For Reporting Diagnostic Accuracy Studies—Artificial Intelligence	These guidelines help to improve transparency and completeness of reporting of diagnostic accuracy studies.
TRIPOD-AI [27]	Development of a reporting guideline for diagnostic and prognostic prediction studies based on artificial intelligence. Transparent Reporting of a multivariable prediction model for Individual Prognosis Or Diagnosis–Aritificial Intelligence	They will both be published to improve the reporting and critical appraisal of prediction model studies that applied ML techniques for diagnosis and prognosis.
PROBAST-AI [27]	Statement and the Prediction model Risk Of Bias ASsessment Tool–Artificial Intelligence	
MI-CLAIM [28]	Minimum Information about CLinical Artificial Intelligence Modeling	This improves the reporting of information regarding clinical AI algorithms.
MINIMAR [29]	MINimum Information for Medical AI Reporting	This establishes the minimum information necessary to understand intended predictions, target populations, hidden biases, and the ability to generalize these emerging technologies.
DECIDE-AI [30]	Developmental and Exploratory Clinical Investigation of DEcision-support systems driven by Artificial Intelligence	This improves the evaluation and reporting of human factors in clinical AI studies. This guideline will address the essential role that human factors will have in how a clinical AI algorithm performs.

The main guidelines regarding the use of AI in clinical research [24] (AI = artificial intelligence; ML = Machine Learning).

## Data Availability

Not applicable.
